# Phase 2, multicenter, open-label study of tigatuzumab (CS-1008), a humanized monoclonal antibody targeting death receptor 5, in combination with gemcitabine in chemotherapy-naive patients with unresectable or metastatic pancreatic cancer

**DOI:** 10.1002/cam4.137

**Published:** 2013-10-25

**Authors:** Andres Forero-Torres, Jeffrey R Infante, David Waterhouse, Lucas Wong, Selwyn Vickers, Edward Arrowsmith, Aiwu Ruth He, Lowell Hart, David Trent, James Wade, Xiaoping Jin, Qiang Wang, TaShara Austin, Michael Rosen, Robert Beckman, Reinhard von Roemeling, Jonathan Greenberg, Mansoor Saleh

**Affiliations:** 1 University of Alabama at BirminghamBirmingham, Alabama; 2 Sarah Cannon Research Institute/Tennessee Oncology, PLLCNashville, Tennessee; 3 Oncology Hematology Care Clinical TrialsCincinnati, Ohio; 4 Scott and White HospitalTemple, Texas; 5 University of MinnesotaMinneapolis, Minnesota; 6 Chattanooga Oncology and Hematology AssociatesChattanooga, Tennessee; 7 Lombardi Comprehensive Cancer Center, Georgetown UniversityWashington, District of Columbia; 8 Florida Cancer SpecialistsFort Meyers, Florida; 9 Virginia Cancer InstituteRichmond, Virginia; 10 Decatur Memorial Hospital, Cancer Care InstituteDecatur, Illinois; 11 Daiichi-Sankyo Pharma DevelopmentEdison, New Jersey; 12 Georgia Cancer SpecialistsAtlanta, Georgia

**Keywords:** Monoclonal antibodies, pancreatic cancer, phase 2, tigatuzumab, TNF-related apoptosis-inducing ligand

## Abstract

Tigatuzumab is the humanized version of the agonistic murine monoclonal antibody TRA-8 that binds to the death receptor 5 and induces apoptosis of human cancer cell lines via the caspase cascade. The combination of tigatuzumab and gemcitabine inhibits tumor growth in murine pancreatic xenografts. This phase 2 trial evaluated the efficacy of tigatuzumab combined with gemcitabine in 62 chemotherapy-naive patients with histologically or cytologically confirmed unresectable or metastatic pancreatic cancer. Patients received intravenous tigatuzumab (8 mg/kg loading dose followed by 3 mg/kg weekly) and gemcitabine (1000 mg/m^2^ once weekly for 3 weeks followed by 1 week of rest) until progressive disease (PD) or unacceptable toxicity occurred. The primary end point was progression-free survival (PFS) at 16 weeks. Secondary end points included objective response rate (ORR) (complete responses plus partial responses), duration of response, and overall survival (OS). Safety of the combination was also evaluated. Mean duration of treatment was 18.48 weeks for tigatuzumab and 17.73 weeks for gemcitabine. The PFS rate at 16 weeks was 52.5% (95% confidence interval [CI], 39.3–64.1%). The ORR was 13.1%; 28 (45.9%) patients had stable disease and 14 (23%) patients had PD. Median PFS was 3.9 months (95% CI, 2.2–5.4 months). Median OS was 8.2 months (95% CI, 5.1–9.6 months). The most common adverse events related to tigatuzumab were nausea (35.5%), fatigue (32.3%), and peripheral edema (19.4%). Tigatuzumab combined with gemcitabine was well tolerated and may be clinically active for the treatment of chemotherapy-naive patients with unresectable or metastatic pancreatic cancer.

## Introduction

Although pancreatic cancer accounts for approximately 3% of all the new cancer cases diagnosed in the United States, it is the fourth leading cause of all cancer deaths [1]. Despite multiple clinical trials with new anticancer agents and more aggressive surgical procedures, the 5-year disease-free survival is 2–9% for those patients diagnosed with locally advanced or metastatic disease [2]. Gemcitabine became the standard treatment of locally advanced or metastatic pancreatic cancer based on an increase in survival over fluorouracil [3]. In 2007, the United States Food and Drug Administration (FDA) approved gemcitabine in combination with erlotinib, a potent epidermal growth factor receptor tyrosine kinase inhibitor, for the treatment of chemotherapy-naive patients with locally advanced, inoperable, or metastatic pancreatic cancer based on the increase in survival by 23% (hazard ratio, 0.82) over gemcitabine alone [4]. However, the long-term survival for advanced disease remained extremely poor, with a median progression-free survival (PFS) at 16 weeks of 44% for patients treated with gemcitabine alone and 51% for those treated with gemcitabine in combination with other drugs (such as chemotherapy or erlotinib) and a median overall survival (OS) of only 6–8 months [3–10]. The FOLFIRINOX combination of chemotherapy (irinotecan, folinic acid, 5-fluorouracil, and oxaliplatin) showed improvement in PFS and OS compared with gemcitabine alone (PFS: 6.4 vs. 3.3 months; OS: 11.1 vs. 6.8 months, respectively) but there was a considerable increase in toxicity, which precludes the use of the regimen in all patients [11]. Most recently, the combination of gemcitabine and *nab*-paclitaxel improved OS and PFS compared with gemcitabine alone (OS: median 8.5 vs. 6.7 months and PFS: median 5.5 vs. 3.7 months, respectively) in the treatment of metastatic pancreatic cancer [12]. Nevertheless, there remains a need for new agents or combinations of agents.

Tumor necrosis factor (TNF)-related apoptosis-inducing ligand (TRAIL), a member of the TNF superfamily of cytokines, is a type 2 membrane protein that is expressed in the majority of normal tissues and can undergo protease cleavage, resulting in a soluble form able to bind to TRAIL death receptors (DRs) [13]. TRAIL induces apoptosis of cancer cells in vitro and has potent tumor activity against tumor xenografts of various cancers in vivo via DRs [13]. Although five receptors for TRAIL have been identified, only two of them (DR4 and DR5) are able to trigger apoptosis of tumor cells through activation of the extrinsic apoptotic pathway (caspase cascade mediated) [13–15]. Interestingly, the other three receptors lack a cytoplasmic death domain and do not mediate apoptosis [16]. High expression of DR5 is frequently observed in various human cancers including colorectal [17, 18], hepatic [19], breast [20], non–small cell lung [21], prostate [22], ovarian [20], and pancreatic [23] cancers.

Tigatuzumab (CS-1008) is the humanized version of the agonistic anti-DR5 murine monoclonal antibody TRA-8 [13–15]. It is composed of the complementarity determining region of the murine monoclonal antibody TRA-8 and the variable region framework and constant regions of human immunoglobulin IgG-1 mAb58'CL [23]. Tigatuzumab induces apoptosis after binding to DR5 in tumor cell lines, resulting in the death of targeted human cancer cells [23, 24]. Tigatuzumab has demonstrated potent in vitro cytotoxic activity against multiple DR5-positive human tumor cell lines, including pancreatic cell lines, and significant in vivo antitumor activity against human tumor xenografts in nude mice with minimal toxicity toward normal tissues [23, 25, 26]. A phase 1, single-agent, dose-escalation study of tigatuzumab in patients with relapsed or refractory carcinomas showed that tigatuzumab is well tolerated with no infusion reactions or grade 3/4 toxicity; the maximal tolerated dose was not reached [27]. In the study, 41% of the patients had stable disease (SD) for a prolonged period of time suggesting antitumor activity. Disease stabilization was observed in patients with hepatocellular carcinoma, carcinoma of the head and neck, colon carcinoma, and cholangiocarcinoma with the duration of stabilization ranging from 81 to 798 days.

The phase 2 study described in this report was designed to evaluate the efficacy of tigatuzumab administered in combination with gemcitabine to chemotherapy-naive patients diagnosed with unresectable or metastatic pancreatic cancer.

## Material and Methods

### Patients

Male and female patients older than 18 years of age with histologically or cytologically confirmed unresectable or metastatic pancreatic cancer that were not previously treated with chemotherapy were enrolled in this clinical trial. All patients had measurable disease at baseline per Response Evaluation Criteria in Solid Tumors (RECIST Version 1.0; http://www.eortc.be/recist/), had a Karnofsky Performance Status (KPS) score ≥60 [28], and had adequate organ and bone marrow function as evidenced by: hemoglobin ≥9.0 g/dL; absolute neutrophil count ≥1.5 × 10^9^/L; a platelet count ≥100 × 10^9^/L; serum creatinine <1.5 mg/dL or creatinine clearance >60 mL/min; aspartate aminotransferase, alanine aminotransferase, and alkaline phosphatase ≤2.5 × the upper limit of normal (ULN) in subjects with no liver metastasis and ≤5.0 × the ULN in subjects with liver metastasis, and total bilirubin ≤2.5 × the ULN. Exclusion criteria included anticipation of a major surgical procedure or radiotherapy during the study; presence of significant cardiovascular disease (New York Heart Association class III or greater); central nervous system involvement; clinically significant active infection that required antibiotic therapy or a history of a positive serology for human immunodeficiency virus; partial or complete bowel obstruction; psychiatric illness that precluded informed consent; and pregnancy and breastfeeding. Patients previously treated with radiation therapy were not excluded.

### Study design and treatment schedule

This was a phase 2, multicenter, single-arm study of tigatuzumab combined with gemcitabine in chemotherapy-naive patients with unresectable or metastatic pancreatic cancer. A treatment cycle was defined as 4 weeks. Patients received tigatuzumab intravenously on days 1, 8, 15, and 21 (8 mg/kg loading dose followed by 3 mg/kg per week) and intravenous gemcitabine on days 1, 8, and 15 (1000 mg/m^2^). Disease was restaged every two cycles (every 8 weeks). Treatment continued without interruption in patients with objective response or SD until progressive disease (PD) or unacceptable toxicity occurred. Patients with PD or unacceptable toxicity were discontinued from study treatment. The planned study duration was 12–18 months. All patients gave informed consent to participate in the study, which was approved by local Institutional Review Boards and conducted in accordance with the ethical principles of the Declaration of Helsinki, International Conference on Harmonisation Guideline E6 for Good Clinical Practice and applicable local regulatory requirements.

### Study end points

The primary efficacy end point was the PFS rate at 16 weeks (PFS rates at 3, 6, 9, and 12 months were also described). Secondary efficacy end points were the objective response rate (ORR) based on RECIST 1.0, duration of response, OS, and safety of the combination. The ORR was defined as the proportion of patients who achieved best overall response of confirmed complete responses (CR) or partial responses (PR). PFS was defined as the time from the date of initial treatment to the date of the first objective documentation of PD or death. OS was defined as the time from the first administration of study drug to the date of death. The duration of response was defined as the time from the date of the first documentation of objective response (i.e., CR or PR) to the date of the first documentation of PD. An unknown response was defined as no tumor assessment after the first infusion of study drug and no recorded clinical disease progression.

Treatment-emergent adverse events (TEAEs) were collected and reported from the time of the first dose administration of the study drug to 30 days after the last dose administration. Toxicities were graded according to National Cancer Institute Common Terminology Criteria for Adverse events, Version 3.0.

Human anti-human antibody measurements (HAHA) were conducted before the infusion of tigatuzumab, at 4 and 8 weeks after the tigatuzumab infusion and every 8 weeks thereafter during active treatment, and wherever possible, 3 months after the end of treatment. A HAHA measurement was also taken at the time when a patient withdrew from the study. The HAHA analysis was performed using a qualitative solid-phase assay designed to detect anti-CS-1008 antibodies in human serum. In this assay, anti-CS-1008 antibodies were captured by CS-1008 bound on a microtiter plate. Captured anti-CS-1008 antibodies were detected with biotin-labeled CS-1008 followed by a commercial streptavidin-horseradish peroxidase (HRP) conjugate. Tetramethylbenzidine was used as the substrate to produce colorimetric optical density. If a sample response was above the established cut-off point (0.040), it was considered potentially positive for the presence of anti-CS-1008 antibodies and further testing was performed. A response below the cut-off point classified the sample as negative for anti-CS-1008 antibodies.

### Statistical analysis

The primary end point of the study was PFS at 16 weeks. Based on published data, the 16-week PFS rate for single-agent gemcitabine was approximately 44%, while it was approximately 51% for the gemcitabine combinations [3–10]. Thus, with 60 patients treated in this trial, the distance between the point estimate and the 95% one-sided lower (upper) bound for the 16-week PFS rate was approximately 10% if the estimate was around 50%. Standard survival analyses using a Kaplan–Meier approach were performed for PFS and OS. The best overall tumor response was summarized. Descriptive statistics (i.e., mean, standard deviation, median, minimum, and maximum) were used in the summary of continuous variables from this trial. Frequency and percentage of observed levels were reported for categorical measures. The per protocol analysis set for efficacy and safety included patients who received at least one dose of tigatuzumab and gemcitabine and who had no major protocol violations.

## Results

### Patients

Between August 2007 and August 2010, a total of 65 patients were enrolled in the study; of these, 61 were included in the efficacy analysis and 62 in the safety analysis only (three patients failed to meet entry criteria and did not receive any treatment per protocol and one patient failed to meet the inclusion criteria but received the study combination). Baseline characteristics of the 61 patients included in the safety and efficacy analysis are listed in Table 1. Thirty-six patients were men and 25 women, with a median age of 61 years (range, 36–78) (Table 1). Fifty-three (87%) patients had metastatic disease, while only 8 (13%) were locally advanced and were not surgical candidates. Patients were predominantly white (95%). The median number of days from diagnosis to initiation of protocol therapy was 22 (range, 4–149). The majority of patients had a KPS ≥80 (KPS 80–90, 74%; KPS 100, 10%). No patient had received prior chemotherapy.

**Table 1:** Demographic and baseline characteristics of all study patients who received tigatuzumab in combination with gemcitabine for metastatic pancreatic cancer and met the inclusion criteria (per protocol analysis set).

	All patients (*N*=61)
Median age (years) (range)	60.6 (36–78)
Gender, *n* (%)	
Male	36 (59)
Female	25 (41)
Race, *n*	
White	58 (95.1)
Black or African American	2 (3.3)
White, Black, or African American	1 (1.6)
Median height (cm) (range)	167.60 (151.1–190.5)
Median weight (kg) (range)	79.10 (44.5–129.1)
Tumor stage at diagnosis, *n* (%)	
Stage IV	53 (86.9)
Nonstage IV	8 (13.1)
Time from diagnosis of pancreatic cancer to study treatment (days)	
Mean	31.6
Standard deviation	25.25
Median	22.0
Range	4–149
Karnofsky Performance Status (KPS) score, *n* (%)	
60	2 (3.3)
70	7 (11.5)
80	19 (31.1)
90	26 (42.6)
100	6 (9.8)
>90	32 (52.5)
Unknown	1

### Efficacy

Sixty-one patients were included in the analyses of efficacy and these results are shown in Table 2. Eleven (18%) patients discontinued study participation before the first scheduled evaluation: two patients withdrew consent (one due to severe nausea, one for nonmedical reasons), four patients had a rapid disease progression, and five patients had serious medical conditions including: stroke (one patient, unrelated), sepsis due to *Clostridium difficile* colitis (one patient, unrelated), perforated bowel (one patient, possibly related to gemcitabine in the opinion of the investigator), and cardiac conditions (two patients, one of them possibly related to gemcitabine in the opinion of the investigator).

**Table 2:** Best overall tumor response for patients who received tigatuzumab in combination with gemcitabine for metastatic pancreatic cancer and met the inclusion criteria (per protocol analysis set).*

Category	All patients (*N*=61)
CR	
*N* (%)	0
95% CI	0.0; 5.9
PR	
*N* (%)	8 (13.1%)
95% CI	5.8; 24.2
Median duration of response days per patient	309days (112, 562, 109, 421, 281, 337, 55, 366)
Objective response (CR or PR)	
*N* (%)	8 (13.1%)
95% CI	5.8; 24.2
SD	
*N* (%)	28 (45.9%)
95% CI	33.1; 59.2
PD	
*N* (%)	14 (23.0%)
95% CI	13.2; 35.5
Inevaluable	
*N* (%)	11 (18.0%)
95% CI	9.4; 30.0

For all subjects, mean duration of treatment was 18.48 (range, 1–88.3)weeks for tigatuzumab and 17.73 (range, 1.0–87.3)weeks for gemcitabine. CR, complete response; PR, partial response; SD, stable disease; PD, progressive disease.

1 Treatment was discontinued at disease progression.

**Figure 1:**
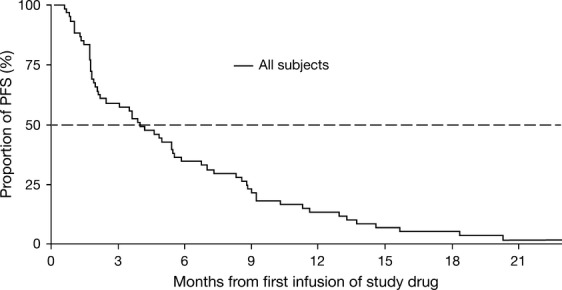
Kaplan–Meier plot of progression-free survival (PFS) for all subjects who received tigatuzumab in combination with gemcitabine for metastatic pancreatic cancer and met the inclusion criteria (per protocol analysis set; *n* = 61). PFS was defined as the time from the date of the first administration of study drug (day 1) to the date of the first objective documentation of disease progression or death resulting from any cause, whichever came first. Overall, 61 patients had disease progression or died.

**Figure 2:**
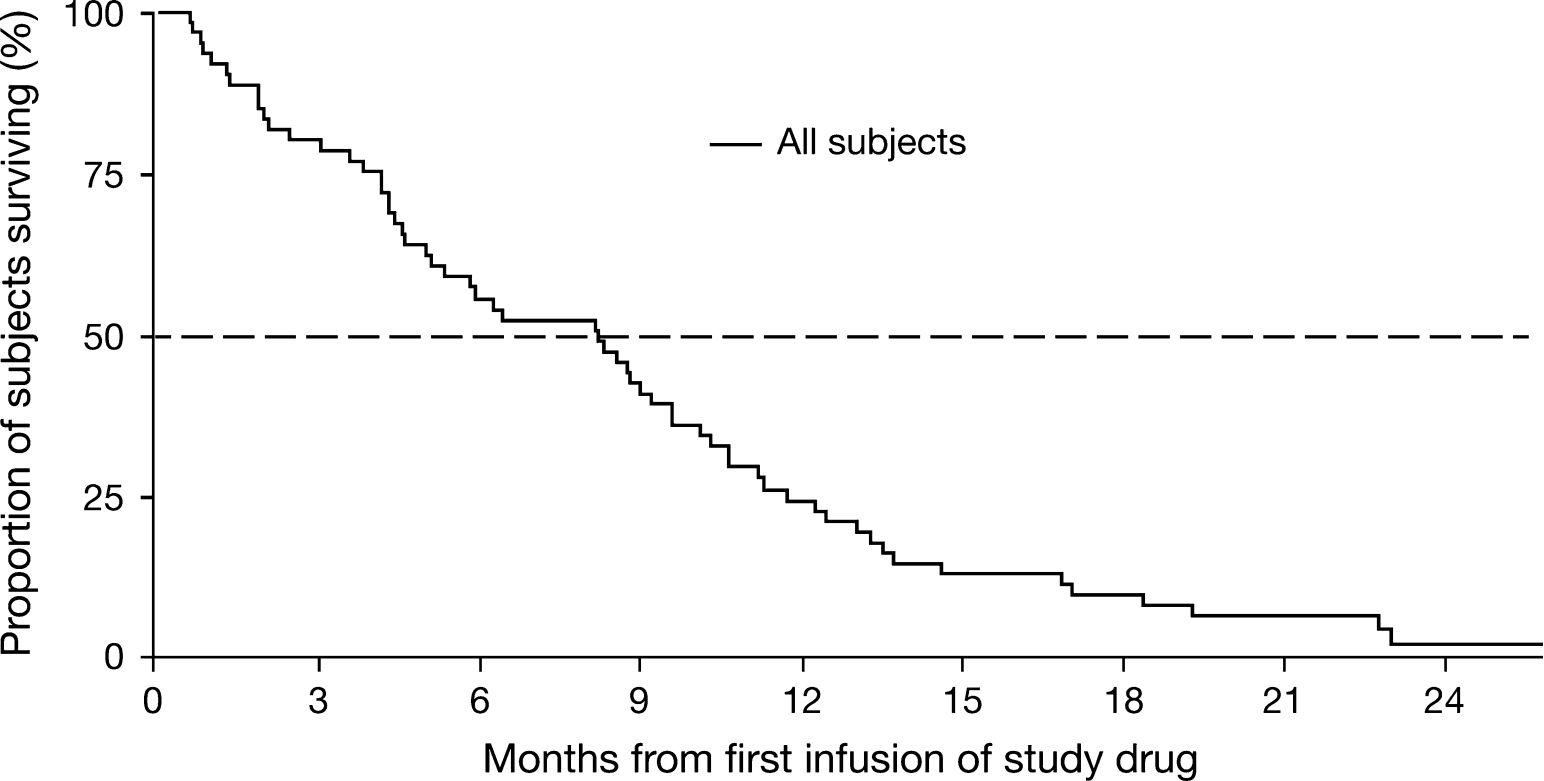
Kaplan–Meier plot of overall survival (OS) for all subjects who received tigatuzumab in combination with gemcitabine for metastatic pancreatic cancer and met the inclusion criteria (per protocol analysis set; *n* = 61). OS was defined as the time from the date of the first administration of study drug (day 1) to the date of death. Overall, 59 subjects died.

The PFS rate was 52.5% (95% confidence interval [CI], 39.3–64.1%) at 16 weeks, 34.4% (95% CI, 22.9–46.3%) at 6 months, 21.3% (95% CI, 12.1–32.2%) at 9 months, and 13.1% (95% CI, 6.1–22.8%) at 1 year. As seen in Figures 1 and 2, the median PFS was 3.9 months (95% CI, 2.2–5.4 months), and the median OS was 8.2 months (95% CI, 5.1–9.6 months), respectively. The OS rate was 55.7% (95% CI, 42.4–67.1%) at 6 months, 24.6% at 1 year (95% CI, 14.7–35.9%), and 13.1% at 15 months (95% CI, 6.1–22.8%). The ORR (CR + PR) was 13.1% (eight patients, all PR). In addition, 28 (45.9%) patients had SD, and 14 (23%) patients had PD. The median duration of response for those patients that achieved a PR was 309 days (mean, 280.4; range, 55–562 days; Table 2).

### Safety and tolerability

As described previously, 62 patients, who received at least one dose of the agents used in the trial, were included in the safety analysis of the study. For all subjects, the mean duration of treatment was 18.48 weeks for tigatuzumab and 17.73 weeks for gemcitabine. Tigatuzumab dose was not modified in the trial. Table 3 illustrates the adverse events observed in at least 20% of the patients independent of the relation to protocol medications. As can be seen in the table, the majority of the adverse events were grade 1, 2, and 3 (75.8%); only four grade 4 adverse events were seen and no grade 5 adverse events were observed. Sixty-nine percent of the adverse events were reported by the investigators as possibly related to tigatuzumab and 87.1% were reported as possibly related to gemcitabine. The most common adverse events were nausea (75.8%), fatigue (69.4%), abdominal pain (51.6%), constipation (50%), fever (48.4%) peripheral edema (40.3%), diarrhea (38.7%), anorexia (35.5%), and anemia (33.9%).

**Table 3:** Summary of treatment-emergent adverse events (TEAEs) and serious TEAEs experienced by >20% of subjects who received tigatuzumab in combination with gemcitabine for metastatic pancreatic cancer (safety analysis set; *n*=62).

	Adverse events seen in more than 20% of the patients, *n* (%)					
	
	All	Toxicity grade				
	
		1	2	3	4	5
Nausea	47 (75.8)	22 (35.5)	21 (33.9)	4 (6.5)	0	0
Fatigue	43 (69.4)	13 (21)	24 (38.7)	6 (9.7)	0	0
Abdominal pain	32 (51.6)	15 (24.2)	8 (12.9)	8 (12.9)	1 (1.6)	0
Constipation	31 (50)	20 (32.3)	9 (14.5)	2 (3.2)	0	0
Fever	30 (48.4)	19 (30.6)	9 (14.5)	2 (3.2)	0	0
Peripheral edema	25 (40.3)	16 (25.8)	8 (12.9)	1 (1.6)	0	0
Diarrhea	24 (38.7)	17 (27.4)	5 (8.1)	2 (3.2)	0	0
Anorexia	22 (35.5)	10 (16.1)	12 (19.3)	0	0	0
Anemia	21 (33.9)	7 (11.3)	8 (12.9)	5 (8.1)	1 (1.6)	0
Anxiety	18 (29)	10 (16.1)	7 (11.3)	1 (1.6)	0	0
Asthenia	17 (27.4)	6 (9.7)	8 (12.9)	3 (4.8)	0	0
Dyspnea	16 (25.8)	7 (11.3)	1 (1.6)	6 (9.7)	2 (3.2)	0
Back pain	15 (24.2)	7 (11.3)	6 (9.7)	2 (3.2)	0	0
Neutropenia	15 (24.2)	2 (3.2)	2 (3.2)	11 (17.7)	0	0
Insomnia	15 (24.2)	12 (19.3)	3 (4.8)	0	0	0
Headache	14 (22.6)	9 (14.5)	4 (6.5)	1 (1.6)	0	0
Thrombocytopenia	14 (22.6)	5 (8.1)	4 (6.5)	5 (8.1)	0	0
Dehydration	14 (22.6)	2 (3.2)	5 (8.1)	7 (11.3)	0	0
Weight loss	13 (21)	9 (14.5)	4 (6.5)	0	0	0

Thirty-five (56.5%) patients experienced serious adverse events while on therapy; nine were related to the protocol medications and 26 related to disease. Of the nine treatment-related events, one was reported by the investigator as tigatuzumab related (peripheral edema) and the other eight were considered by investigators as possibly related to gemcitabine (pleural effusion, urinary tract infection, pneumonia, anemia [two patients], hemolytic uremic syndrome with bowel perforation, heart failure, and vomiting). Six (9.7%) patients died during the study; one case was considered by the investigator as possibly related to gemcitabine and unrelated to tigatuzumab (congestive heart failure); one case was assessed as unrelated to tigatuzumab (causality assessment for gemcitabine not reported) while the other four deaths were considered secondary to disease progression.

Thirty-three (53.2%) patients discontinued treatment due to disease progression, 13 (21.0%) due to adverse events, three (4.8%) withdrew consent, and 13 (21.0%) discontinued for other reasons. Of the patients who discontinued study therapy due to an adverse event, one was possibly related to tigatuzumab (peripheral edema) while two were possibly related to gemcitabine (hemolytic uremic syndrome, congestive cardiac failure). None of the patients developed human antihuman antibodies during the trial.

## Discussion

Despite advances in the treatment of cancer over the last decade, pancreatic cancer continues to have a poor prognosis due to the lack of effective agents; thus, new therapeutic strategies are needed. In this phase 2 study, a total of 62 untreated patients with unresectable or metastatic pancreatic cancer were treated with the standard therapeutic agent gemcitabine combined with tigatuzumab, based on encouraging preclinical data that showed a synergistic effect between the chemotherapy agent and the monoclonal antibody [23, 25, 26], and based on the clinical safety demonstrated by the monoclonal antibody in the phase 1 clinical trial [27]. For all patients, the mean duration of treatment was 18.48 weeks for tigatuzumab and 17.73 weeks for gemcitabine.

The primary end point of this study, PFS rate at 16 weeks, was 52.5%, which is similar to that seen in published studies using gemcitabine alone (median 44%; range 33–51) or in combination with other agents including the FDA-approved agent erlotinib (median 51%; range 45–60) [4–10]. In addition, our study demonstrated a median OS of 8.2 months, which is comparable to that seen in previous studies of patients with advanced pancreatic cancer treated with gemcitabine alone or gemcitabine plus other agents; with a median OS of 3.6–6.8 months and 3.8–11.1 months, respectively [3, 4, 6, 11, 29]. Of note, in the two pivotal gemcitabine trials, the OS was 3.9 [30] and 5.7 months [3], while OS was 6.4 months in the pivotal trial of gemcitabine plus erlotinib [4]. In the FOLFIRINOX trial, median OS was 11.1 months versus 6.8 months with gemcitabine alone; however, FOLFIRINOX was associated with increased toxicity [11] and in the recent phase 3 MPACT trial, median OS was 8.5 months with *nab*-paclitaxel plus gemcitabine [12]. Other efficacy parameters observed in this study were also comparable with previous studies using gemcitabine alone or in combination, including percent of patients with PR or SD and duration of response [3, 4, 6, 11, 29, 30].

Thus, the numeric trends described in the current study compared with historical data observed with gemcitabine alone suggest a possible contribution of tigatuzumab to the antitumor efficacy of gemcitabine. However, the current study represents a single-arm trial, and no definitive conclusions can be drawn regarding the contribution of tigatuzumab to the observed ORR, PFS, or OS data.

Tigatuzumab was safe and well tolerated when administered in combination with gemcitabine. The most commonly reported adverse events were nausea, fatigue, vomiting, abdominal pain, and pyrexia, which are typical of disease progression in this patient population and are similar to those reported in previous studies of gemcitabine [3, 4, 29]. The majority of tigatuzumab-related adverse events were grade 1 or grade 2. No patient had a grade 4 or grade 5 adverse event that was related to tigatuzumab treatment. Only one patient had a serious adverse event (peripheral edema) that was considered by the investigator to be related to tigatuzumab treatment.

In conclusion, data from this phase 2 clinical trial show that the combination of tigatuzumab and gemcitabine is well tolerated and may be clinically active in the treatment of chemotherapy-naive patients with unresectable or metastatic pancreatic cancer.
